# Indigenous Burning as Conservation Practice: Neotropical Savanna Recovery amid Agribusiness Deforestation in Central Brazil

**DOI:** 10.1371/journal.pone.0081226

**Published:** 2013-12-11

**Authors:** James R. Welch, Eduardo S. Brondízio, Scott S. Hetrick, Carlos E. A. Coimbra

**Affiliations:** 1 Escola Nacional de Saúde Pública, Fundação Oswaldo Cruz, Rio de Janeiro, Rio de Janeiro, Brazil; 2 Department of Anthropology, Indiana University, Bloomington, Indiana, United States of America; 3 Anthropological Center for Training and Research on Global Environmental Change, Indiana University, Bloomington, Indiana, United States of America; University of Western Ontario, Canada

## Abstract

International efforts to address climate change by reducing tropical deforestation increasingly rely on indigenous reserves as conservation units and indigenous peoples as strategic partners. Considered win-win situations where global conservation measures also contribute to cultural preservation, such alliances also frame indigenous peoples in diverse ecological settings with the responsibility to offset global carbon budgets through fire suppression based on the presumed positive value of non-alteration of tropical landscapes. Anthropogenic fire associated with indigenous ceremonial and collective hunting practices in the Neotropical savannas (cerrado) of Central Brazil is routinely represented in public and scientific conservation discourse as a cause of deforestation and increased CO_2_ emissions despite a lack of supporting evidence. We evaluate this claim for the Xavante people of Pimentel Barbosa Indigenous Reserve, Brazil. Building upon 23 years of longitudinal interdisciplinary research in the area, we used multi-temporal spatial analyses to compare land cover change under indigenous and agribusiness management over the last four decades (1973–2010) and quantify the contemporary Xavante burning regime contributing to observed patterns based on a four year sample at the end of this sequence (2007–2010). The overall proportion of deforested land remained stable inside the reserve (0.6%) but increased sharply outside (1.5% to 26.0%). Vegetation recovery occurred where reserve boundary adjustments transferred lands previously deforested by agribusiness to indigenous management. Periodic traditional burning by the Xavante had a large spatial distribution but repeated burning in consecutive years was restricted. Our results suggest a need to reassess overreaching conservation narratives about the purported destructiveness of indigenous anthropogenic fire in the cerrado. The real challenge to conservation in the fire-adapted cerrado biome is the long-term sustainability of indigenous lands and other tropical conservation islands increasingly subsumed by agribusiness expansion rather than the localized subsistence practices of indigenous and other traditional peoples.

## Introduction

Efforts to reduce the negative environmental and biodiversity impacts of commercial agriculture and pasture activities are increasingly recognized to benefit from locally based knowledge and practices [1]. The new United Nations Intergovernmental Science-Policy Platform on Biodiversity and Ecosystem Services (IPBES) program, for instance, recognizes explicitly the need to include local and indigenous knowledge as part of its assessment and policy reach mandates [2]. Nevertheless, international conservation discourse generates important misconceptions by presuming the destructiveness of human alteration of tropical landscapes and, specifically, overgeneralizing about the effects of anthropogenic fire in diverse cultural and ecological settings.

Indigenous reserves and other types of protected areas have become the most important policy mechanism for controlling deforestation [3], [4] and fire [5] associated with the booming expansion of large-scale agriculture in Northern and Central Brazil. Today these areas represent a mosaic of conservation islands amid expanding monoculture landscapes and are often considered win-win situations where global conservation goals and reduction in carbon emissions also contribute to cultural preservation [3], [6], [7]. Nevertheless, the generalized association between fire impacts on conservation, CO_2_ emissions, and climate change is contributing to important misrepresentations when extrapolated without attention to regional and local contexts.

Increasingly part of the widespread implementation of Reducing Emissions from Deforestation and Forest Degradation (REDD) and REDD+ programs [8], these ideas regarding anthropogenic fire are also widely incorporated by interest groups as political narratives contesting indigenous rights to land in Brazil. They promote the notion that indigenous burning activities represent a destructive mentality (“culturally endorsed pyromania,” to borrow an expression applied to indigenous practices in northern Australia [9]) out of line with the conservation agenda set forward at global, national, and local levels to revert biodiversity losses and climate change. Thus, localized traditional landscape management practices are subjected to the scrutiny of unexpected alliances (e.g., large commercial ranchers using conservation arguments). Increasingly, indigenous and other traditional peoples in Brazil are framed with the responsibility to offset a substantial portion of the national and global carbon budget through conservation stewardship and REDD+ projects financed by high-emission industrialized countries [10]. Nevertheless, industrial agriculture and ranching expansion since the late 1960s has transformed the Central Brazilian cerrado into one of the most threatened biomes in the country [11], [12].

### Biodiversity and Fire in the Cerrado

The cerrado, included among the 35 most important “hotspots” in the world [13], [14], is a diverse but highly threatened tropical savanna-like biome covering over 2 million hectares, approximately 24% of the total area of Brazil, mainly in the Central Brazilian Plateau. According to one estimate, of the approximately 10,000 plant species identified in the cerrado, about 4,400 are endemic [15]. The agricultural potential of the cerrado and its positive economic benefits for Brazil have been widely publicized for decades [16]. At least 35% of the cerrado region has been completely converted to intensive human use since 1960, predominantly for pasture, intensive monoculture and, more recently, eucalyptus cultivation [11]. Recent land conversion to soybean and sugarcane biofuel production in the cerrado results in large carbon debts estimated to require 17 to 37 years to repay [17]. In lieu of buffering the Amazon from further expansion of agricultural commodities and biofuels, in coming years the cerrado region is prone to increasing pressures with associated climatic impacts [18], [19] and escalation of conflicts between agribusiness and indigenous interests [20].

Differently from the Brazilian rainforests – Amazonian and Atlantic forests, adaptation to periodic surface fires is an evolutionary characteristic of cerrado ecology independently from human action [21], [22]. Lightning, which is common during the rainy season, is considered the principal environmental ignition factor in the cerrado [23] and explains evidence of cerrado wildfires as early as 32,000 BP, long before human presence in the region [24], [25]. The recurrence of fire in a determined cerrado landscape is thought to have resulted in over 70% of the total cerrado biomass being subterranean, as well as other anatomical adaptations providing resistance to fire damage and favoring vigorous vegetation regrowth immediately after the fires, even before first rains [21], [26], [27]. The diversity of structural forms in the cerrado biome is considerable. Depending on the environmental factors that predominate in a given region, such as soil depth and drainage, level of acidity, and aluminum saturation, vegetation can range from dense forests to open grasslands, with many intermediate configurations in close proximity [27].

The presence and potential positive ecological effects of anthropogenic fire in the cerrado has been noted by botanists travelling through the region since the nineteenth century. In 1892, botanist Eugene Warming observed that plant form and vegetation structure in the cerrados of Minas Gerais, Brazil, benefitted from fire, with most plants undergoing vigorous sprouting and flowering shortly after being burned [28]. As he wrote, “the most beautiful, also the most blooming and freshest green field I have ever seen was precisely one that was burned in October” [28]. More recently, based on observations of cerrados in Xavante territory, botanist George Eiten wrote that frequencies of indigenous fires greater than every one or two years had no evident long-term effects on plant physiognomy and recently burned areas attracted game animals through the creation of mineral licks and the abundant production of tender plant shoots [27].

Recent ecological studies show that cerrado landscapes may be even more highly resistant to the impacts of fire, including anthropogenic burning, than previously thought [21], [22]. Also, fire regimes that vary in intensity, frequency, and seasonality may have significantly different effects on biomass and vegetation structure [26]. Periodic burning has been found to result in less intense and more fragmented, and therefore less destructive, fires than occur in protected cerrado areas where fire suppression causes the accumulation of large amounts of dead aboveground biomass [22], [23], [29]. Conversely, sporadic fires produce positive effects on cerrado biodiversity [25], [30]. According to botanists, fires benefit cerrado plants due to adaptations such as fire-dependent sexual reproduction and dispersal, as well as rapid regeneration and increased flowering and germination [29], [30]. These adaptations in turn benefit wildlife through increased availability of such foods as pollens, nectars, shoots, and fruits [29].

Although fire can effect major short term landscape change through its impact on cerrado vegetation, the resilience of fauna populations is indicated by scant evidence of local animal extinctions [22], [31]. Additionally, the low temperatures and patchy distribution of cerrado surface fires allow animals ample escape routes and areas of cover. Ecological evidence also shows that moderate intensity fires increase the availability of foods for some taxa, including small vertebrates and arthropods, by stimulating vegetative growth, flowering, and fruiting [32]–[34]. Ecological studies specifically evaluating the impacts of indigenous burning activities in the cerrado also found no evidence that animal populations, including those of economically important game species, decreased following fires [35]–[39].

### Cultural Aspects of Hunting with Fire

Hunting with fire is a characteristic subsistence activity of many indigenous inhabitants of the Central Brazilian cerrado, including such Gê-speaking indigenous groups as the Kayapó, Krahô, Canela, and Xavante [25]. Contrary to journalistic representations of Xavante hunting with fire as a cause of cerrado degradation and deforestation [40], [41] or an impediment to vegetation recovery [42], ethnographic accounts indicate periodic burning by these indigenous peoples has minimal or desirable effects on cerrado vegetation structure and productivity [43], [44]. Our previous ethnographic research [45] revealed that the Xavante people, eastern Mato Grosso State, use fire for hunting annually during the dry season (primarily July to September and less frequently the moister months of May and June) in widely dispersed locations throughout the reserve.

**Figure 1:**
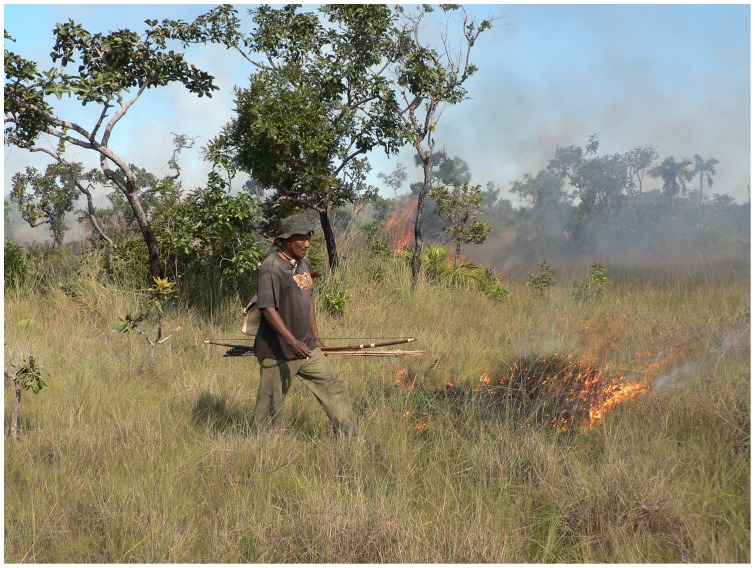
Xavante elder during collective hunt with fire, Pimentel Barbosa Indigenous Reserve, Brazil, 2005. Photograph by James R. Welch. The legal representative of the depicted individual provided informed written consent for the publication of his image.

Xavante collective hunting with fire often occurs in conjunction with such ceremonial occasions as weddings and adolescent rites of passage [45]. Hunting excursions follow a ritualized format, whereby representatives from opposite exogamous moieties set fire to the vegetation as they run footraces in two wide arcs towards a distant finishing place. As the fire expands in a shifting mosaic pattern, numerous hunters act individually or in collaboration with other hunters to dispatch fleeing game animals (Figure 1). At the conclusion of hunting excursions, the acquired game meat is often pooled and ceremoniously presented to designated recipients, such as in-laws and initiation leaders. From the Xavante perspective, collective hunting with fire is an essential component of the ceremonial activities that distinguish them ethnically and causes no undesirable ecological changes when practiced according to traditional protocols. To the contrary, Xavante cultural knowledge recognizes that traditional periodic burning accelerates the vegetative growth of some plants, increases the availability of certain animal foods, and reduces the intensity of future fires.

Although the Xavante practice agriculture and in the 1970s sold a small portion of rice yields as part of a government development project, today this activity is largely restricted to domestic production in small rotating patches, primarily in narrow gallery forests along river margins [46]. In contrast to agriculture, periodic hunting with fire has a large spatial distribution and therefore greater potential for causing landscape-scale ecological effects within reserve boundaries. However, Xavante strategies for hunting with fire are highly regulated by specific traditional protocols and knowledge aimed to maximize both short-term hunting productivity and long-term sustainability. Our ethnographic research [45] shows that when planning fires, Xavante hunters discuss such diverse factors as season, weather, winds, soil and foliage moisture, type of vegetation, and natural barriers in order to control their intensity, spatial distribution, and frequency through time (see also [47]–[49]). Among these considerations ample attention is given to the potential negative ecological effects of burning the same patches of vegetation in consecutive years, especially during extended droughts.

### Aims of this study

In this paper, we evaluate conservation narratives regarding the relative long-term ecological impacts of indigenous and agribusiness management of Brazilian neotropical savanna landscapes based on estimates of diachronic environmental changes and indigenous burning patterns in a cerrado landscape in Mato Grosso State. Specifically, we compared land cover change over four decades inside and within a 20 km buffer area around Pimentel Barbosa Indigenous Reserve (Xavante ethnic group) in order to ascertain their respective deforestation patterns. Multi-temporal spatial analyses of changes in vegetation cover (1973–2010) were performed utilizing satellite and georeferenced field data, as well as archival research for reconstructing changes in reserve boundaries since 1972. Additionally, in order to evaluate the contemporary Xavante burning regime contributing to observed results at the end of this sequence, we analyzed the spatial and temporal distribution of anthropogenic fire within the reserve utilizing a four year sample (2007–2010). This integrated study expands upon our long-term ethnographic and interdisciplinary research carried out in collaboration with the Xavante people since the 1990s [45], [50]–[53].

## Methods

### Ethics statement

Permission for the project was provided by the National Indian Foundation (FUNAI) and the National Ethics Committee (CONEP, permission #652-2011).

### Location and population

Research was conducted in the Pimentel Barbosa Indigenous Reserve, Mato Grosso State, Brazil. The majority of this territory pertains to the Araguaia River Basin, although a small portion at the western boundary pertains to the Xingu River Basin. The ecological biome is cerrado, with a division occurring between western portions of the reserve, in which cerrado *sensu stricto* and dry forests predominate, and eastern portions, in which inundated grasslands are more prevalent. The boundaries of this reserve underwent a series of alterations during the study period (through decrees in 1972, 1975, and 1979, and ratification in 1986).

The 2010 population of Pimentel Barbosa Indigenous Reserve was approximately 1,400 individuals residing in nine villages [45]. The entire population pertains to the Xavante ethnic group and all individuals speak the Xavante language (Gê language family).

### Georeferenced field data

Field data was collected for 117 georeferenced training samples inside and outside the reserve. These included multiple locations for 16 land cover classes (water, bare soil, agricultural field/pasture, fire scar, open grassland, arboreous (*murundu*) grassland, scrub grassland, cerrado *sensu stricto*, dense cerrado, rupicolous cerrado, *cerradão* woodland, dry upland forest, gallery forest, bamboo forest, *buriti* palm forest, and *ipuca* flooded forest). Data collected included vegetation type, land cover, land use history, regrowth status, notable species, and photographs. Elder members of the indigenous community accompanied all fieldwork activities and assisted with the identification of land use histories at all training sample locations.

### Multi-temporal remote sensing: classification and deforestation analysis

Using Landsat MSS (WRS I Path/Row –240/69; 240/70; 80 meter spatial resolution) and TM (WRS II Path Row –223/69; 223/70; 224/69; 224/70; 30 meter spatial resolution) imagery from the United States Geological Survey (USGS) and Brazilian National Institute for Space Research (INPE), a multi-temporal data set was established for the image dates 1973, 1986, 2000, and 2010. Each date was created using a mosaic of two (1973) and four (1986, 2000, 2010) contiguous images. All images were subjected to radiometric and atmospheric calibrations, georeferenced, and image subsets were layer stacked to form multitemporal images. Image classification was carried out using ERDAS IMAGINE 11.0.4. Deforestation analysis utilized a land cover classification system consisting of water, bare soil/agricultural, grassland/cerrado, and forest.

Thematic data extraction for reserve boundaries and a 20 km buffer zone around the reserve was carried out using the tabulate area tool in ArcGIS 10.1. Classification procedure for the 1973 image was based on unsupervised procedures only; for 1986, 2000, and 2010 classification was based on a hybrid approach (i.e., involving integration of unsupervised and supervised classifiers). Images were independently classified. Image classification benefited from field collected georeferenced training samples. Field sites representative of natural vegetation were used as references for all dates, while recently altered sites were used as references for 2010. A layer of field sites was created and referenced into the multi-temporal image dataset.

Unsupervised analysis using ISODATA started with high-dimensional unsupervised clustering (50 classes) of the whole image area. Classes were analyzed according to spectral-structural differences (relationship between spectral data and indicators of vegetation structure), spatial distribution, and statistical values (mean, standard deviation, and covariance) for all spectral bands except thermal. Using supervised procedures, training samples with known vegetation and history of land use were created and used to develop spectral signatures for the classes of interest in each data set for 1986, 2000, and 2010. Training samples and unsupervised classes were combined into a single spectral signature file. Analysis of training samples and unsupervised signatures include a combination of Transformed Divergence separability analysis, spectral signature comparison, and correlation analysis of spectral-vegetation data. Based on these procedures, signatures were merged (or eliminated) to produce a final signature set representing a gradient of vegetation structure, from bare soil/agricultural to high forest. Signature sets were submitted to a probability-based Gaussian Maximum-Likelihood classifier. Classification accuracy of the 2010 classified image was assessed for aggregated classes (i.e., bare soil/agricultural, grassland/cerrado, and forests) used in transition matrix analysis. A set of 30 test fields of known features (i.e., training samples not used for supervised classification) was used to assess the accuracy of each class. Classification accuracy ranged from 91% for bare soil/agricultural, 86% for grassland/cerrado, and 84% for forests. Confusion between classes occurred mainly in areas of transitional vegetation structure with mixed elements of soil, herbaceous, arbustive, and arboreal coverage.

Legal descriptions of historical reserve boundaries (dates 1972–1975, 1975–1979, 1979–1986, and 1986-present) were digitally reconstructed from archival maps and descriptive documents of delimiting landmarks and overlaid on the multi-temporal image dataset in ArcGIS. Analyses of deforestation trajectories were developed by calculating transition matrices in ERDAS IMAGINE for each subsequent pair of images starting with the earliest date (1973–1986, 1986–2000, and 2000–2010). For the transition matrix analysis grassland and cerrado classes were aggregated. Deforestation was considered when a shrub or arboreous class converted into bare soil or agricultural fields/pasture) at a subsequent date. Areas of bare soil/agricultural that returned into grassland/cerrado or forest classes were recoded as such to indicate a reforestation process. The final thematic layer produced eight classes, including those that remained unaltered across dates (i.e., water, bare soil/agricultural, grassland/cerrado, and forest) and those involving change, including deforestation before 1973, deforestation between 1973 and 1986, deforestation between 1986 and 2000, and deforestation between 2000 and 2010. The final layer was recoded to show the area deforested for each period. We used cross-tabulation in ArcGIS to allow estimation of deforestation during each period within (respective reserve boundaries) and outside (respective buffer zones) the reserve.

We used ancillary data to verify the expansion of agriculture and pasture within the reserve during the late 1970s, years not represented in the analysis sequence described above but described by Xavante observers and historical sources as the apex of agribusiness encroachment and deforestation in and around Pimentel Barbosa Indigenous Reserve [45], [50]. A thematic map of land cover for 1979 was produced based on cartographic maps by the Brazilian Institute for Geography and Statistics (IBGE) printed at a scale of 1∶100,000 [54]. These IBGE maps were developed from aerial photographs of the area from 1967 and fieldwork from 1979. The map was digitized to produce a thematic layer, which was overlaid to a Landsat MSS image (1976) for correction. This map discriminates cerrado and areas opened for agriculture and pasture. The resulting thematic layer provides important supporting evidence for the deforestation of cerrado areas within reserve boundaries and the buffer zone towards the end of the 1970s.

### Fire matrix analysis

Burned areas within reserve boundaries in the years 2007–2010 were mapped using Landsat TM and ETM+ imagery from the United States Geological Survey (USGS). Images free of clouds were acquired for each month from July-September for each of the four years to capture the primary annual burning season. As with the land-cover classifications, the imagery used for burned area mapping were coregistered to ensure spatial accuracy. Each image was classified using the ISODATA clustering algorithm in ERDAS IMAGINE. Fifty clusters were initially created using a convergence threshold of 0.995. Burned areas were isolated from other land covers through spectral signature analysis and visual interpretation. Mixed pixels indicating burning scars were manually digitized if necessary. Classifications from all months in the same year were then mosaicked and manually cleaned before running a 3×3 majority filter to complete the burned area classification. Each pair of years was subjected to a transition matrix analysis to estimate areas of consecutive burnings: first the transition from 2007 to 2008, second the transition from 2007/2008 to 2009, and third the transition from 2007/2008/2009 to 2010. A final transition matrix image 2007–2010 comprised of 5 classes was created to illustrate land area by number of consecutive years burned (i.e., burning in no more than 0, 1, 2, 3, and 4 consecutive years).

## Results

**Figure 2:**
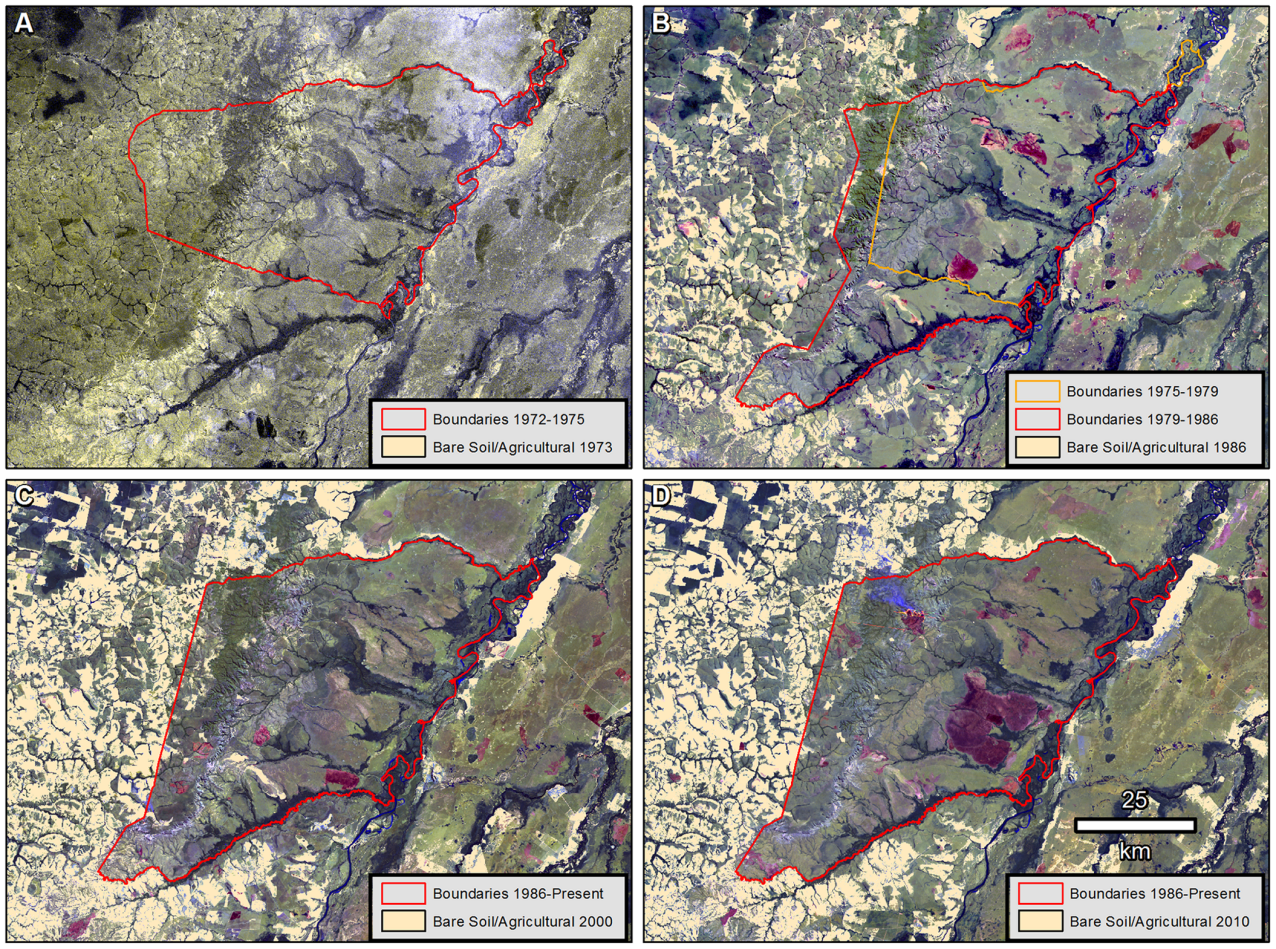
Mosaic of calibrated satellite imagery overlaid with reserve boundaries and deforestation mapped from 1973 to 2010, Pimentel Barbosa Indigenous Reserve, Brazil, 1973–2010. Landsat MSS and TM imagery used in our analyses was obtained courtesy of the United States Geological Survey (USGS) and from the Brazilian National Institute for Space Research (INPE) under Creative Commons Attribution-ShareAlike 3.0 Unported License.

The evolution of reserve boundaries overlaid on deforestation maps for 1973, 1986, 2000, and 2010 show the progression of deforestation and vegetational recovery inside and outside the reserve (Figure 2). Deforestation outside the reserve indicates a pattern of encroachment from the west with its eastern limit shifting through time as reserve boundaries were adjusted. Comparison of intermediate reserve boundaries (Figure 2B:1975–1979 and 1979–1986) indicate that most bare soil and agricultural fields or pasture inside the reserve in the years 1986 and 2000 was in locations previously outside or bordering the reserve limits. Additionally, large deforested patches evident inside the reserve in 1986 and 2000, especially near its western and southern boundaries, were greatly reduced in size by 2010.

**Figure 3:**
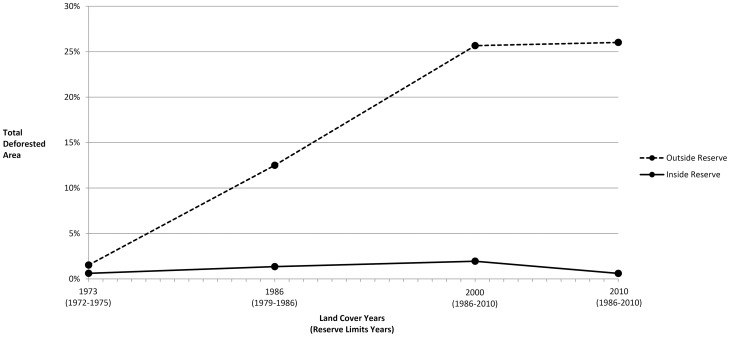
Total deforested area (%) relative to total area inside and outside (20 km buffer) corresponding limits of Pimentel Barbosa Indigenous Reserve, Brazil, 1973–2010.

Considering the historical reserve boundaries associated with each image year (Figure 3), the deforested area inside the reserve remained stable at 0.6% between 1973 (1,645.9 ha) and 2010 (1,989.0 ha). However, intermediate years showed higher proportions (1.3% in 1986 and 1.9% in 2000) associated with land previously impacted by agricultural operations being incorporated into the reserve and, to a lesser extent, with agricultural projects promoted by the federal government inside the reserve. The deforested area inside the reserve decreased by 68.9% (4,412.5 ha) from its peak in 2000 to the end of the sequence in 2010. The proportion of deforested land in a 20 km buffer area outside historical reserve limits increased continually from 1.5% (9,589.6 ha) in 1973 to 26.0% (175,412.4 ha) in 2010, with the largest increase observed in the period 1973–1986 (718.4%; 76,470.8 ha) and the smallest in 2000–2010 (1.4%; 2,383.4 ha).

**Figure 4:**
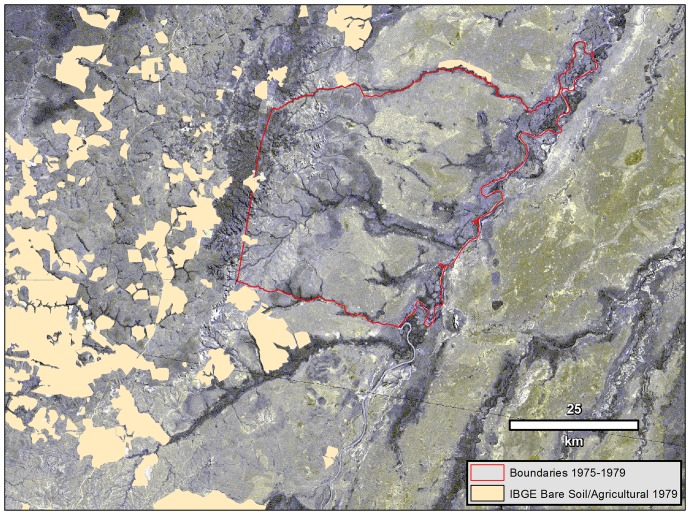
Mosaic of calibrated satellite imagery (1976) overlaid with reserve boundaries (1975–1979) and areas of bare soil used for livestock pasture and agricultural fields (1979), Pimentel Barbosa Indigenous Reserve, Brazil. Landsat MSS imagery used in our analyses was obtained from the Brazilian National Institute for Space Research (INPE) under Creative Commons Attribution-ShareAlike 3.0 Unported License. Cartographic data were digitized with permission from IBGE topographic maps [54].

Data from Brazilian topographic maps based on 1979 aerial photography (Figure 4) identify the distribution of commercial livestock pasture and agricultural fields when reserve boundaries were most restricted (1975–1979). These boundaries are also shown without temporally corresponding satellite imagery in Figure 2B. Figure 4 shows that many of the locations first appearing as bare soil/agricultural within the reserve in 1986 (Figure 2B) were in fact previously deforested by agribusiness before they were incorporated into the reserve by legal changes to the reserve limits.

**Figure 5:**
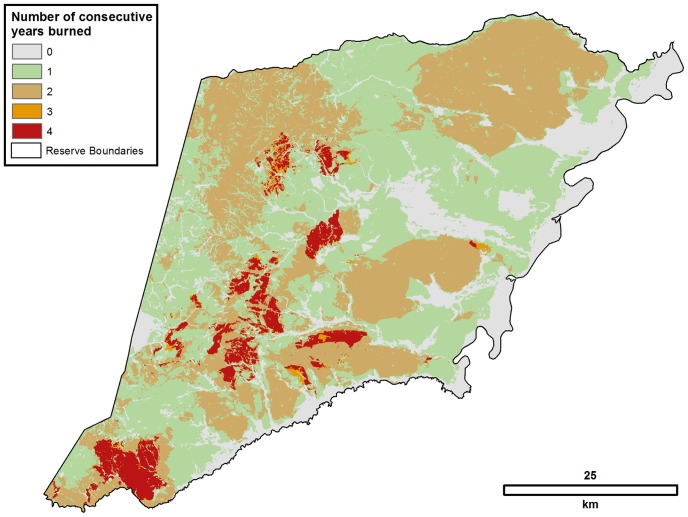
Burn scar transition matrix analysis showing land area by number of sequential years burned, Pimentel Barbosa Indigenous Reserve, Brazil, 2007–2010. Landsat TM and ETM+ imagery used in our analyses were obtained courtesy of the United States Geological Survey (USGS).

Vegetation burning matrix analyses show the distribution of lands inside the reserve by number of consecutive years burned from 2007 to 2010 (Figure 5). The majority of the reserve burned at least once during the four-year period (83.2%; 273,979.26 ha) but only 40.7% (134,043.84) burned in two or more consecutive years. The largest areas were burned one (42.5%; 139,935.42 ha) or two (35.2%; 115,829.01 ha) years sequentially. Only 0.6% (2,019.24 ha) and 4.9% (16,195.59 ha) of the reserve was burned in three and four consecutive years, respectively. Additionally, 16.8% (55,496.52 ha) did not burn in any year.

### Implications for cerrado conservation

**Figure 6:**
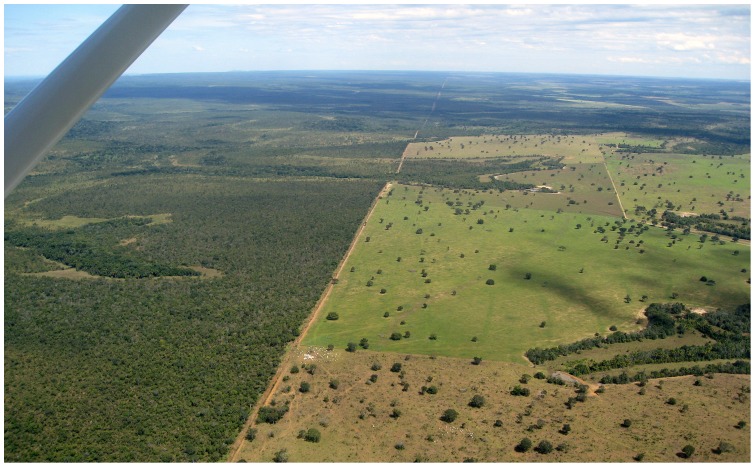
Agribusiness deforestation (right) along western border of Pimentel Barbosa Indigenous Reserve (left), Brazil, 2009. Photograph by James R. Welch.

The proportion of deforested land inside reserve boundaries increased in 1973–1986 and 1986–2000, but decreased in 2000–2010. Considering the entire period 1973–2010, the proportion of deforestation remained constant at an extremely low level (0.6%) inside the reserve. Most deforestation inside the reserve occurred along the western and southern boundaries, which suffered the greatest impacts of regional eastward economic expansion since the mid-1970s (Figure 6).

A notable factor in this progression is the evolution of reserve boundaries through time. In particular, Xavante political action [45] resulted in substantial increases in the reserve size (62.5% between 1975 and 1986), mainly through reclaiming traditional territory from adjacent agribusiness lands to the west and south. By returning these areas to indigenous management, previously deforested lands subsequently underwent vegetational recovery, thereby restoring the proportion of deforested land inside the reserve to the value observed four decades earlier (0.6%). This interpretation is corroborated by evidence from topographic maps generated in 1979, when the reserve was reduced to its smallest size, showing extensive agribusiness deforestation in areas which were subsequently incorporated or reincorporated into the reserve. A substantial portion of these areas were still classified as bare soil/agricultural in 1986 and 2000 but returned to grassland/cerrado or forest by 2010.

In contrast, outside reserve boundaries the proportion of deforested land increased in all periods, reaching 26% in 2010. The comparatively smaller increase observed in 2000–2010 contrasts with a regional pattern of rapid agribusiness expansion in the same period [55]. This difference may be attributable to decreased economic advantage of agricultural expansion along the western boundary of Pimentel Barbosa Indigenous Reserve given the extensive availability of lands nearby that were less intensively deforested before 2000.

The temporal burn scar pattern observed during the annual burning seasons of the last four years of this four decade sequence (2007 to 2010) indicates that almost 60% of the reserve was never burned or not burned in consecutive years. Only a very small portion of the reserve, about 5%, was burned in three or more consecutive years. Comparison of Figures 2D and 5 reveals that vegetation cover was maintained or recovered even in areas of high fire periodicity within the reserve. Thus, the observed Xavante burning pattern involved predominantly low fire periodicity and did not cause deforestation even where fires occurred in multiple consecutive years. Although these data do not allow us to draw direct comparisons with non-anthropogenic cerrado fire regimes, differences are likely to occur in terms of seasonality and frequency. In particular, whereas we observed the Xavante hunt with fire most often during the dry season, most cerrado fires not ignited through human agency are caused by lightning during rainy or transitional seasons [23], [29], [56]. However, in general terms, both scenarios involve highly varied surface fire periodicity and patchy distributions.

Ecological studies demonstrate that periodic cerrado fires can have positive effects on species diversity, as well as other environmental measures, and ought to be considered a strategic technique for environmental management in this biome [21], [22], [25], [26], [29], [30]. This practice has yet to be incorporated in environmental management policies by the Brazilian government as it is in some other parts of the world with fire-adapted landscapes [57]–[59]. This oversight may result from a tendency for the Brazilian rainforests, which are not fire adapted, to receive more attention from conservationists and environmental policies than the cerrado biome and to frame narratives of conservation and climate change mitigation in Brazil.

**Figure 7:**
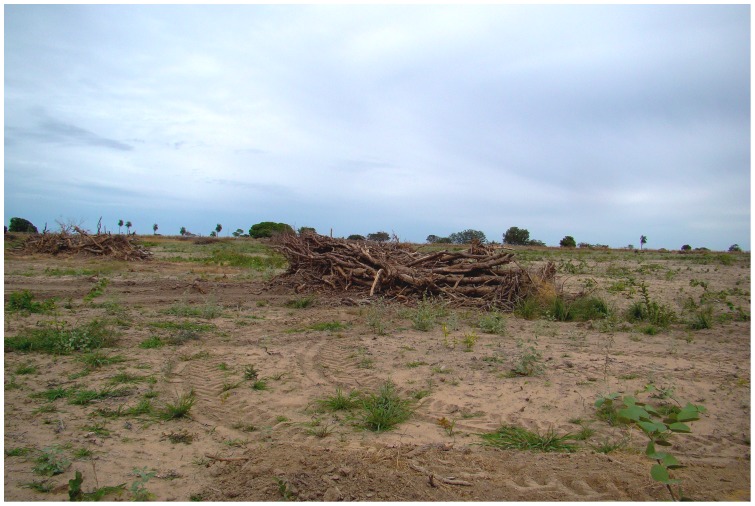
Remains of mechanized tree-chaining on private property adjacent to western boundary of Pimentel Barbosa Indigenous Reserve, Brazil, 2012. Photograph by Carlos E. A. Coimbra Jr.

Additionally, some scientific research on climate change [60] conflates rainforests with cerrados, despite their enormous ecological distinctions, because they are both included in Brazil's geopolitical division “Legal Amazon” (*Amazônia Legal*). However, the cerrado landscape and the indigenous reserves in Central Brazil face different threats than the Brazilian rainforests due to their distinct economic potentials for commercial agricultural development and ecological responses to anthropogenic fire [3], [15], [22], [61]. For the past 50 years, the cerrado has been massively exploited through large scale clearing techniques largely absent in the Brazilian rainforests, involving mechanized tree-chaining that uproots trees in such a manner that regrowth is completely averted (Figure 7). Conversely, commercial exploitation of Amazonian rainforests (in some cases involving indigenous peoples) involves some deforestation activities that are less frequent in the cerrado of Central Brazil, including illegal timber logging, large-scale industrial burning, and gold mining. As the Brazilian government and the agribusiness sector respond to international pressures to curb carbon emission from deforestation in the Amazonian rainforests, economic pressure on the cerrado and associated forest clearing activities tend to increase.

Much of policy and public discourse on tropical conservation presumes the positive value of non-alteration of tropical landscapes. For example, although international initiatives such as REDD and REDD+ promote the inclusion of indigenous peoples and their interests in adopting policies for reducing carbon emissions, these programs nevertheless often aim at halting traditional subsistence activities presumed to be responsible for CO_2_ emissions, irrespective of their particular social and ecological contexts [62]. Similarly, scientific conservation discourse in Brazil often assumes that anthropogenic burning in the cerrado, whether practiced by indigenous peoples or others, is uniformly destructive, contributes to global warming, and threatens the value of indigenous reserves and other conservation areas [63]–[68]. However, as demonstrated by the results of the present study, indigenous Xavante landscape management practices over four decades, including periodic collective hunting with fire and political advocacy for federal recognition of traditional lands, maintained the integrity of the cerrado landscape and sustained vegetational recovery as compared to adjacent lands under non-indigenous management.

Our findings call into question the widespread assumption, evident in international conservation narratives such as those associated with the implementation of REDD and REDD+ programs, that the non-alteration of tropical landscapes through the suppression of indigenous anthropogenic burning is a beneficial strategy for reducing deforestation in indigenous reserves in the cerrado biome. This point is especially pertinent because rapid plant regrowth in the cerrado quickly re-assimilates carbon emissions from appropriate fire regimes [22], [26]. Furthermore, our results suggest a need to reassess the implications of overreaching conservation and reduction in carbon emission narratives, increasingly shared by contrasting interest groups in Brazil and elsewhere, about the purportedly destructive nature of indigenous land use, particularly landscape management with fire [40], [41]. In this context, the real challenge of cerrado conservation is to address the long-term sustainability of ‘conservation islands,’ such as indigenous reserves, and the carbon offset approach to deal with the inter-connected nature of land use systems that now characterizes Brazil and elsewhere, whereby conservation units are increasingly subsumed by the impacts of the larger landscapes to which they pertain.
